# American Public Health Problems

**Published:** 1915-10

**Authors:** 


**American Public Health Problems.** Mortality from Accidents, by age and sex, United States registration area, 1903-1912, (Rate per 100,000 of population):

| Ages | Males Rate | Females Rate |
|---|---|---|
| Under 5 | 147.3 | 111.4 |
| 5-14 | 60.3 | 21.4 |
| 15-44 | 131.0 | 18.4 |
| 45 and over | 196.6 | 84.8 |

